# Reconstructing a typology of approaches to navigating diversity: a comparative qualitative study of German nursing teams

**DOI:** 10.1186/s12912-026-04854-y

**Published:** 2026-06-09

**Authors:** Lisa Peppler, Martin Feißt, Maja Apelt, Liane Schenk

**Affiliations:** 1 Charité – Universitätsmedizin Berlin, Corporate Member of Freie Universität Berlin and Humboldt-Universität zu Berlin, Institute of Medical Sociology and Rehabilitation Science, Charitéplatz 1, 10117 Berlin, Germany; 2 Faculty of Economics and Social Sciences, University of Potsdam, August-Bebel-Str. 89, 14482 Potsdam, Germany; 3 Independent Researcher, Am Feuerschanzengraben 14, 37083 Göttingen, Germany; 4 Metaplan - Gesellschaft für Planung und Organisation mbH, Goethestraße 16, 25451 Quickborn, Germany

**Keywords:** Nurse, Diversity, Team, Management, Qualitative research, Social identity theory

## Abstract

**Background:**

Germany faces a severe nursing shortage, increasingly addressed by international recruitment. This shift results in growing socio-cultural and educational diversity within nursing teams. While the experiences of migrant nurses are well-documented, how receiving teams manage this diversity in daily practice remains under-researched. This study investigates team-level patterns of navigating diversity and identifies factors influencing successful integration.

**Methods:**

A qualitative, comparative case study design was employed across six nursing teams (four hospitals, two nursing homes) in Germany. Teams were selected through purposive sampling based on high levels of socio-cultural and educational diversity. Data collection (March 2022–August 2023) included 203 h of participant observation, 25 semi-structured interviews, and 8 focus groups with both nursing management and non-managerial nursing staff focusing on daily cooperation, professional and socio-cultural differences, and team integration processes. Inductive analysis followed the documentary method to reconstruct implicit orientation patterns. Social Identity Theory (SIT) served as the theoretical framework for describing the different team dynamics.

**Results:**

Three distinct team-level approaches to diversity were identified: (1) Routine-based Teams normalise diversity within a stable framework, valuing individual competencies as a natural asset and seamlessly integrating differences into everyday practices. (2) Transition-based Teams actively engage with shifting team configurations through supportive structures but experience operational ambivalence as newly developing learning processes occasionally collide with ingrained routines. (3) Friction-based Teams exhibit a defensive, problem-oriented stance toward diversity, characterised by rigid boundary maintenance, perceived competence conflicts, and a tendency toward social fragmentation under structural strain. Key influencing factors include the facility’s location and patienthood and the role of nursing management.

**Conclusions:**

Successful team collaboration depends on fostering a shared professional framework that recognises and utilises diverse individual competencies. Nursing management must provide stable resources for reciprocal knowledge transfer and actively address structural barriers regarding the valuation of international qualifications. To ensure high-quality care and sustainable staff retention, managerial interventions should implement targeted support measures tailored to the specific dynamics of a team type.

**Clinical trial number:**

Not applicable.

**Supplementary Information:**

The online version contains supplementary material available at 10.1186/s12912-026-04854-y.

## Introduction

Germany, like many Global North countries, faces a critical shortage of skilled labour in the care sector due to demographic change. In 2019, the total number of nursing professionals employed in Germany stood at 1.62 million [1]. However, since 2022, employment growth in German nursing has been sustained exclusively by international healthcare professionals [2]. By the year 2049, Germany is projected to face a shortage of between 280,000 and 690,000 nursing staff [1]. This situation has driven political efforts to promote the recruitment of international nursing staff since 2012, positioning Germany as an emerging player in active recruitment despite a late start in establishing the structured integration programs seen in the UK or Australia [3]. As a result, Germany has become the third most significant destination country for nurse migration [4]. From 2013 to 2023, the employment of non-German citizens in the care sector increased by over 250% [2]. In 2021, 9.6% of nurses in Germany were non-German citizens, a number that is rapidly growing [5, 6]. This trend leads to increasing diversity within nursing teams with regard to cultural and educational backgrounds. Consequently, inpatient facilities must support nursing staff in managing this growing diversity, including language barriers, skill variations, and differing professional self-perceptions, in their daily work.

Many countries make a similar distinction between nursing activities that are carried out by less qualified personnel and those that are reserved for skilled professionals. The specific activities and demarcations vary from country to country, but in most cases there are differences between different qualification levels in nursing with corresponding areas of responsibility. In contrast, in Germany, the responsibility for both treatment and basic care lies with skilled nursing staff, while nursing assistants support them in providing basic care. At the time the data was collected, German recruitment efforts were aimed exclusively at skilled nursing staff; it was only the reforms of the Skilled Immigration Act (2023/24) that extended access to the German labour market to nursing assistants from other countries. The integration of these international nursing professionals into the German labour market requires a formal recognition procedure. During this procedure, foreign-trained nursing professionals usually work as nursing assistants in German institutions [6].

International research on nurse migration is extensive, as evidenced by numerous reviews [7–9]. Most qualitative studies focus on the individual experiences of migrant nurses in the host country [10, 11]. Key challenges highlighted are consistent across countries, primarily revolving around language and communication barriers, cultural differences, experiences of discrimination, and the process of recognising foreign qualifications [8, 12].

While the challenges of integrating international nurses are well-documented, recent research also identifies key facilitating factors for successful cooperation. These include structured onboarding processes, targeted language support, and, crucially, a proactive organisational culture that fosters mutual learning [13–15]. Successful integration practices often rely on ‘bridge-builders’ within teams and leadership styles that actively address and mediate socio-cultural differences [13, 16]. However, empirical evidence remains fragmented regarding how these factors interact within specific nursing teams to ensure long-term cohesion.

However, while the individual perspective of migrant nurses has been well-documented, the receiving nursing teams have been treated rather implicitly [17, 18]. Only gradually is research focusing on how multicultural nursing teams are managed [16, 19]. Nevertheless, there remains a significant research gap concerning how established inpatient care teams deal with the cultural and educational diversity resulting from international nursing migration in their day-to-day work. Given the global nursing shortage and increasing reliance on international recruitment, understanding team-level dynamics is critical for retention and quality of care.

Therefore, this article aims to narrow the research gap by means of a comparative case study in six nursing teams. We reconstruct three distinct ways in which these teams navigate cultural and educational diversity, and we identify the conditions that positively or negatively influence these team processes. The core research questions guiding this study are: How do nursing care teams deal with their socio-cultural and educational diversity? How can care teams achieve a positive approach to diversity for smooth daily work? How can management support teams in this process?

To elucidate how increasingly diverse nursing teams navigate differences and maintain internal cohesion, this study draws on Social Identity Theory (SIT) [20], which provides a robust social-psychological framework for understanding how individuals categorise themselves and others into social groups and how these categorisations impact team dynamics and intergroup behaviour. The theory posits that people derive a part of their self-concept – their social identity – from belonging to specific groups. Driven by a motivation to maintain a positive distinctiveness, individuals engage in social comparison to evaluate their own group (in-group) positively against others (out-group), which can lead to in-group bias and out-group differentiation. It is crucial to note that the resulting in-groups and out-groups do not necessarily reflect the formal work unit or team structure; they are based on self-categorisation along salient differences. Therefore, this framework is highly pertinent for analysing diverse nursing teams, which are characterised by multiple, overlapping categorisations that influence team processes.

In this study, ‘diversity’ is conceptualised as a multi-dimensional and relational construct. Following a broad definition of diversity, we focus specifically on the intersection of two primary axes:

- Socio-cultural diversity (including migration background, linguistic repertoire, and country of origin), which has been shown to shape team processes and to function as both asset and liability in culturally diverse nursing teams [21].
- Qualification-related diversity (i.e., professional backgrounds, foreign degrees, and levels of formal training), resonating with intersectional analyses that link social categories to different opportunities and constraints in healthcare work [22].

Utilising the theoretical framework of SIT, we conceptualise socio-cultural variations and qualification-related attributes as indicators of social categorisation, which in turn exert an influence on team cohesion. These dimensions are regarded as categories that are constantly negotiated within the teams’ daily routines.

Accordingly, ‘successful integration’ is defined analytically as collaborative inclusion. This dynamic process entails a reduction in the salience of distinct out-group attributes, thereby fostering a shared professional ‘we-orientation’. Hence, ‘success’ is conceptualised as the transition from a problem-oriented perception of differences toward a resource-oriented synergy. This shift is indicative of the development of a shared in-group identity, wherein diverse backgrounds are effectively integrated into the collective work practice.

Furthermore, our study also includes a comparative analysis of two types of inpatient care settings – hospitals and care homes. By taking into account the respective organisational contexts in relation to staff members’ social identities, the SIT offers a valuable approach for identifying the resulting team-specific patterns in dealing with diversity.

## Methods

The objective of this study was to analyse the implicit orientation patterns and collective practices of nursing teams in managing socio-cultural and educational diversity within the context of international nurse migration. The comparative case study design provided the structural framework to capture diverse team dynamics across different organisational contexts. The documentary method was employed as an inductive analytic tool to reconstruct the teams’ implicit, practical knowledge without pre-existing theoretical assumptions. During the comparative analysis of the six cases, typical patterns emerged that distinguished the care teams from one another. This led to the development of the typology presented here, which had not been apparent at the outset; the development of a typology was not part of the initial study design. To adequately represent these inductively reconstructed team types, we opted for SIT as an interpretative lens, which allows for an in-depth explanation of the interaction dynamics and the identified orientation patterns.

### Study design and setting

This qualitative comparative case study was conducted as part of a larger mixed-methods study investigating change processes in German nursing. The ‘case’ is defined as the nursing team, conceptualised as a collective space of experience. From a methodological perspective, teams are regarded as formally regulated action units. The shared professional practice and identical structural conditions of these units result in the formation of collective, implicit knowledge. Our research focuses on how nursing teams manage the intersection of socio-cultural background (migration) and educational background (qualification) within their daily work. Given that diversity is a socially constructed phenomenon whose meaning is context-dependent, we adopted a qualitative approach to reconstruct the actors’ subjective interpretations and the underlying social processes.

The research was conducted in six nursing teams between March 2022 and August 2023 in a major German city, encompassing four teams in hospitals and two teams in nursing homes. A recent publication based on focus groups from this and a previous study examines the transition processes in German nursing due to immigration from abroad and academisation in Germany [23]. In contrast, this article focuses on how nursing teams deal with cultural diversity and foreign academic qualifications. In addition, the research basis in this article is expanded to include semi-structured interviews and participant observation.

The study’s multimethod design follows a logic of complementary triangulation, where each data source contributes a specific analytical layer to the case (the nursing team): participant observation was used to reconstruct the praxeological reality and implicit routines of the teams in their daily work environment. Focus groups were utilised to reconstruct the collective orientation within the team. This method allows for the identification of shared, often pre-reflexive knowledge that guides the team as a social unit. Semi-structured interviews were conducted to capture the individual perspectives of the team members. This integration enables a multifaceted analysis, with observations capturing the ‘performative’ dimension (action), focus groups unveiling the ‘discursive’ collective norms (shared values), and interviews emphasising individual subjective experiences.

### Sampling and recruitment

The study employed criterion-based purposive sampling to ensure a high-density empirical basis for comparing different professional and biographical constellations within nursing teams. The selection of the six care teams was conducted simultaneously and followed the intersection of two key dimensions of diversity:

1. Educational and qualification background; teams were selected to include a mix of staff with.
  - vocational training in Germany,
  - academic degrees obtained in Germany, and.
  - academic degrees obtained abroad.
2. Socio-cultural background and migration experience; sampling focused on teams integrating individuals with.
  - no migration background,
  - a migration background but trained within the German system, and.
  - direct migration experience as international healthcare professionals.

The identification of the teams followed an active recruitment strategy through direct outreach to healthcare facilities. Teams were screened for eligibility through consultations with nursing directorates. A team was considered eligible only if it featured the specific intersectional profile of qualifications and migration biographies required by the research question. Table 1 provides an overview of the key parameters and contextual profiles of the six investigated nursing teams.

**Table 1:** Key structural parameters and contextual profiles of the investigated cases

Team (case)	Institution & Setting	Unit Type / Sector	Team constellation
A	hospital, inner-city	acute care	large-scale (30 + nurses), leavers: retirement, educational leave
B	hospital, inner-city	acute care	medium-scale (10–25 nurses), leavers: retirement, educational leave
C	hospital, peripheral urban	acute care	medium-scale (10–25 nurses), leavers: retirement
D	hospital, peripheral urban	acute care	medium-scale (10–25 nurses), leavers: retirement
E	nursing Home, inner-city	long-term care	medium-scale (10–25 nurses), leavers: retirement, intra-organisational mobility
F	nursing home, peripheral urban	long-term care	large-scale (30 + nurses), rather no leavers (developmental phase)

The total number of six cases was determined at the outset based on the feasibility and scope of the study. Due to the exhaustive and multifaceted character of the data collection process, which encompassed observations, interviews, and focus groups for each case, the designated sample size was sufficient to facilitate the requisite depth of analysis.

Participants were recruited personally by LP and MF with support from team leaders for organisational purposes where necessary. Inclusion criteria for participants comprised all practicing nursing staff, while individuals under the age of 18 were excluded from the study. Sampling aimed for the greatest possible variation across the diversity dimensions of migration and qualification. Table 2 provides an overview of the socio-demographic data of all participants. The sample reflects a broad global spectrum, with participants originating from North and South America, Southeast and Southwest Asia, and various European and African countries.

**Table 2:** Socio-demographic data of participants

Dimension of diversity	Focus groups (*n* = 36)	Semi-structured interviews (*n* = 25)
**Migration experience**		
As an adult	9	7
None or as a child	27	18
**Qualification**		
Foreign trained academic	7	5
German-trained academic	2	6
German-trained vocational	27	14
**Age**		
18–39 years	17	14
40–60 + years	19	11
**Gender**		
Female	32	18
Male	4	7

### Data collection

The data collection employed an iterative-circular research process, linking sequential data collection with ongoing analysis. Data collection was conducted on-site at the respective facilities. Each case study started with participant observation, followed by semi-structured interviews and focus groups.

LP and MF conducted 203 h of participant observation across early and late shifts, focusing on ward routines outside patient rooms, including handovers, team meetings and informal interactions in corridors and break rooms. For legal and ethical reasons, they did not participate in nursing activities or provide hands-on support, but limited their involvement to conversational interaction with staff and remained outside clinical decision-making and hierarchical structures. The duration of observations was distributed as 49 h in Team A, 42 h in Team B, 24 h in Team C, 25 h in Team D, 32 h in Team E and 31 h in Team F. The lower number of hours in Teams C and D was due to temporary COVID‑19 related access restrictions, meaning that only the minimum observation time specified in the study protocol could be realised, whereas the extended field time in Teams A, B, E and F reflects particularly favourable access conditions and a high level of staff motivation to participate in the study. Observations, including spoken interactions, were recorded anonymously by hand and later transferred to structured protocols for data analysis.

25 semi-structured interviews with managerial and non-managerial nursing staff captured individual perspectives on teamwork. The interviews amounted to a total of approximately 21 h, with a mean duration of 58 min. Guidelines were specifically developed for this study based on initial participant observation. Interviews were conducted by MF in German or English, depending on the participants’ preference and language proficiency. The interviews were recorded with participant consent and professionally transcribed. English versions of the interview guides are provided as Additional Files 1 and 2.

LP conducted eight focus groups to capture the collective perspectives of team members. A standardised discussion stimulus – a fictitious scene from everyday care work – was used. The focus groups amounted to a total of approximately 7.5 h, with a mean duration of nearly 60 min. The discussions were recorded with participant consent and professionally transcribed. Focus groups were conducted in German, as this was the only common language among participants, reflecting the linguistic diversity of the teams. In order to guarantee the inclusion of staff members with a variety of linguistic backgrounds, the focus group discussions were adapted into plain German when necessary, corresponding to levels B1 and B2. Crucially, the choice of language was also a deliberate part of the analytical focus. Since the study aimed to investigate team dynamics, the way the teams handled varying levels of German proficiency during the discussions provided valuable data. The extent to which native-speaking colleagues supported their international peers was directly incorporated into the analysis. In this sense, the linguistic challenges encountered during the focus groups acted as a microcosm of daily professional interactions, offering deep insights into the inclusive or exclusionary nature of each team’s identity.

In this study, the adequacy of the empirical basis was assessed using the concept of theoretical sufficiency [24] at three levels. At case level, sufficiency was reached when the triangulation of observations, focus groups and interviews yielded a coherent, non‑contradictory reconstruction of each team’s specific ‘space of experience’; for example, in Team E, later data repeatedly confirmed exclusionary narratives and prejudices towards new international colleagues that had already emerged in earlier interviews, focus group discussions and observations of unsupportive behaviour during joint tasks. At comparative level, sufficiency was achieved when cross‑case analysis of the six diverse team constellations no longer produced additional dimensions beyond the two core axes of the typology, namely collective orientation towards diversity and degree of mutual support. At typology level, theoretical sufficiency was assumed once each of the three types was represented by at least two empirically rich cases and further comparisons did not reveal new combinations of the two dimensions but could be clearly assigned to one of the existing orientation patterns, while still allowing for within‑type heterogeneity, particularly between the two Transition‑based Teams (D and F), which ranged from cautious, often unrealised reflections to proactively supported integration and mutual learning.

**Fig. 1:**
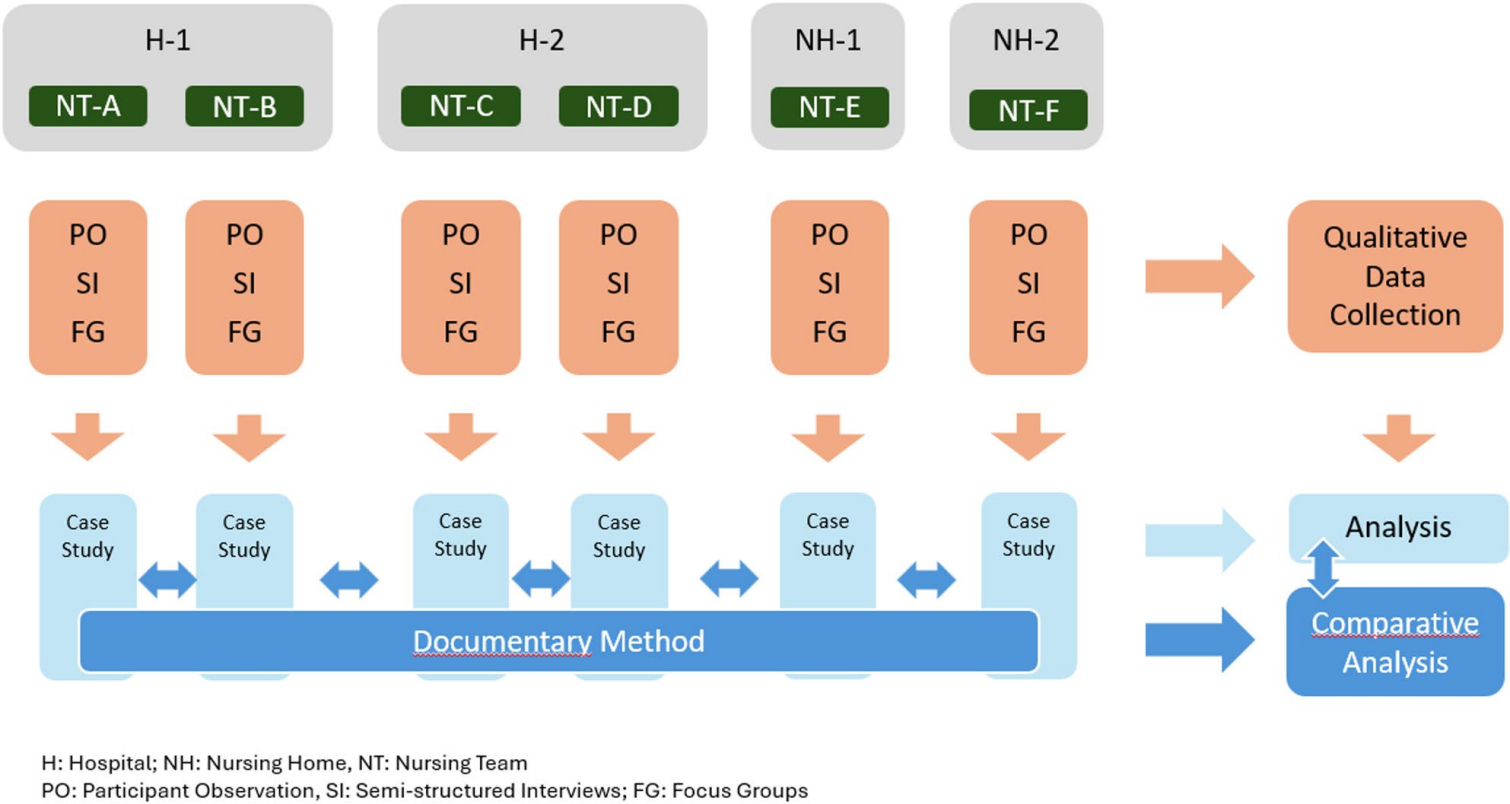
Iterative-circular research process

Figure 1 provides a visual overview of the study’s multi-level research design. It illustrates the integration of the three data collection methods and their alignment with the two institutional settings. This schematic representation serves as a structural map for the subsequent detailed analysis of the six cases.

### Data analysis

For the analysis, all primary data were imported into MAXQDA (2022), including the transcripts of the focus groups, the semi‑structured interviews and the structured protocols from participant observation. These protocols were treated as a praxeological layer that was systematically compared with the discursive material from interviews and focus groups in each case. The data were analysed using the documentary method [25] to reconstruct implicit orientation patterns – the unconscious, shared knowledge and action-guiding mechanisms of everyday team activities.

**Fig. 2:**
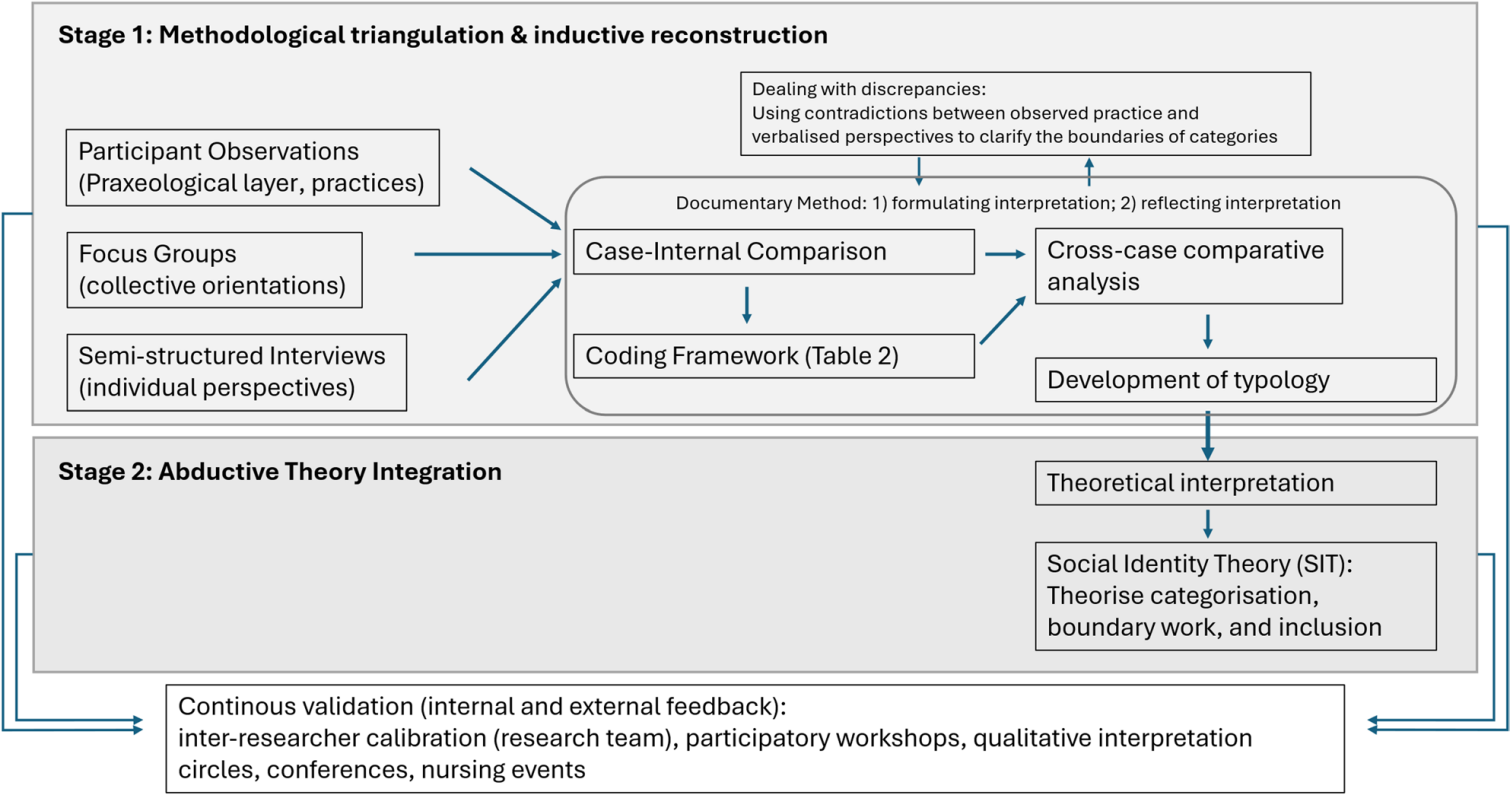
Project-specific analytical workflow: from multi-source methodological triangulation to abductive theory integration and multi-stakeholder validation

The analytical process followed an abductive logic in a two-stage procedure, as illustrated in Fig. 2.

In a first stage, we reconstructed orientation patterns and developed the typology *inductively* using the documentary method. This proceeded in two main steps [25]:

1. Formulating interpretation: transcripts and protocols were summarised to gain an initial overview, identify central group themes, and work out typical communication patterns.
2. Reflective interpretation: relevant passages were selected and subjected to in-depth analysis to reconstruct the underlying, implicit assumptions and orientations that structure the teams’ actions.

A key empirical insight that emerged during this phase was a recurring discrepancy between the formal organisational units (administrative wards) and the subjectively perceived ‘felt’ teams (who was considered a ‘real’ member of the team). The specific ways in which nursing teams navigated this tension became the defining characteristic of the different types.

The coding process was systematically documented within MAXQDA. To ensure a robust audit trail, we maintained memos on the development of interpretive categories and on the decision rules used during the transition from raw data to the final typology [26]. In line with the reconstructive logic of the documentary method, our coding framework was designed to be non-exclusive, allowing for the multidimensionality of nursing practice to be captured [27]. The coding framework combined case-related and thematic dimensions. At the case level, we used team-specific codes (e.g. “Team A” – “Team F”) to capture the particular constellation and development of each nursing team. Thematically, codes covered several interrelated domains of nursing practice and team dynamics, as summarised in Table 3.

**Table 3:** Overview of the coding framework

Main Domain	Sub-codes / Example codes	Code Frequency
Case & Team Identification	Specific team dynamics and contexts (Team A – Team F)	239
Institutional Setting	Specific facility structures (Hospital 1, Hospital 2, Nursing Home 1, Nursing Home 2)	84
Leadership Practices	Management styles, expectations, role of supervisors	182
International Nurses & Migration	Language, onboarding/induction, recognition processes, workplace hierarchies	160
Tasks & Responsibilities	Basic care, treatment care, functional nursing, professional understanding, documentation	212
Interprofessional Collaboration	Cooperation with physicians, cooperation with service staff	150
Qualifications & Trajectories	academic qualifications, vocational qualifications, trainees, care assistants, specific aspects of care for the elderly	96
Macro-Context & Nursing System	Staff shortages, temporary/pool agency nursing, staff turnover, public discourse	94
Socio-demographic Dynamics	Generational aspects, gender dynamics	85
Everyday Workplace Culture	Routine practices, daily work dynamics, informal interactions	90

For example, the focus group statement “Those we have on our ward now, it was very difficult at the beginning because communication was practically non-existent. […] things have settled down very well now because our skilled staff, I’d say, the ones who were already here, they’re really taking them under their wing, so to speak, and showing them a lot.” (F-FG-7) was initially coded as “specific team dynamics – Team F” and “onboarding/induction”. In the comparative analysis, such sequences were grouped under the category “mutual support among team members”, which, in turn, contributed to the reconstruction of the “Transition-based Teams” type.

By systematically comparing case-internal patterns across nursing teams, we identified cross-case commonalities and differences. The resulting three types were distinguished based on two core dimensions:

1. The collective orientation towards socio-cultural and qualification-related diversity (ranging from more resource-oriented to more problem-oriented). The operational criterion for type assignment here was how diversity was praxeologically evaluated in daily routines: empirical indicators for a problem-oriented focus included explicit linguistic partitioning or formalistic isolation during handovers, whereas indicators for a resource-oriented focus manifested as proactive knowledge-sharing and mutual skill integration.
2. The degree of mutual support within the team and from the team leadership. The criteria for type assignment centered on the stability of intra-group boundaries and management style: empirical indicators ranged from active boundary work and managerial distance (resulting in ‘felt’ teams that exclude certain staff members) to universal inclusionary practices and explicit coaching by managers (aligning the formal administrative ward with the subjective team identity).

Only after the empirically grounded typology had been established did we introduce SIT in a second stage as an interpretive lens. SIT was selected because it provides a coherent framework for theorising the observed processes of categorisation and boundary work and for linking the inductively derived team types to more general conceptual debates about group identities and inclusion.

The integration of observations, interviews and focus groups followed the logic of methodological triangulation [28]. Conflicting and converging evidence were handled through step‑by‑step comparative analysis: when observations of daily routines aligned with collective and individual orientations identified in focus groups and interviews, these patterns were treated as highly robust and formed the core of the reconstructed real types. By contrast, divergences – for instance, when individual interviews articulated professional ideals that were contradicted by observed practices – were treated as analytically productive and used to refine both the within‑case interpretations and the emerging typology [28]. Rather than being eliminated as outliers, discrepant data were systematically revisited in team discussions and interpretation groups and used to adjust category boundaries, nuance type descriptions and identify internal tensions within teams.

To ensure analytical rigour, dependability, and credibility throughout the research process, a comprehensive audit trail was maintained using MAXQDA analytical memos to document all meta-decisions and category definitions. Inter-researcher calibration and the minimisation of individual bias were achieved through intensive, almost daily peer debriefings and joint reflections on emerging case interpretations between the primary data collectors (MF and LP). Any initial disagreements between the researchers regarding the assignment of codes or the reconstruction of orientation patterns were systematically discussed within the analytical team until a full interpretive consensus was achieved. To achieve an interpretive consensus and enhance inter-subjective comprehensibility, data were brought into a multi-staged validation framework involving different stakeholders:

A) Internal research team peer-review (weekly). The primary researchers (MF, LP) presented raw case reconstructions to the multi-disciplinary internal team (medical and organisational sociologists, health and cultural scientists, medical students, nursing practitioners). Discussed materials included observation protocols, transcripts, and preliminary case memos. This phase focused on challenging over-interpretations and refining cross-case comparisons. Feedback was integrated and documented as analytical memos directly within MAXQDA.
B) Multi-disciplinary hermeneutic interpretation groups (bi-monthly). Key data sequences were submitted to three independent, external interpretation groups, each comprising five to eight qualitative researchers from organisational sociology, ethnology, health sciences, medicine, and public health. Discussed materials consisted of raw, un-analysed text sequences to uncover blind spots and generate alternative readings. Disagreements were debated until a communicative consensus was reached and documented as analytical memos in MAXQDA.
C) Participatory stakeholder workshops (field-level). Eleven workshops were conducted: six with the participating nursing teams and five with nursing management. Discussed materials consisted of visual summaries and structural maps of the reconstructed categories and orientations. This phase served to actively challenge and nuance our findings. Insights directly informed and refined both the single-case interpretations and the subsequent cross-case comparative analysis.
D) Expert and academic community audits (conferences). The developing typology model was presented and debated at international academic conferences and professional healthcare events with academic peers, nursing staff, and healthcare executives. This external feedback was utilised to sharpen the theoretical extensions, clarify the study’s meso-level contribution, and verify the practical resonance of the three distinct team types within the broader healthcare landscape.

### Reflexivity and researcher positionality

The study was conducted by an equally partnered, multi-disciplinary team: MF, an organisational sociologist specialising in healthcare, and LP, a cultural scientist focusing on health professional migration. The two researchers have extensive experience in qualitative research and methodology. The equal involvement of both researchers throughout the entire field phase allowed for a continuous ‘internal triangulation’ of perspectives. This approach combined an analytical focus on institutional hierarchies (sociological) with a deep sensitivity to cultural practices and narratives (cultural-scientific). Furthermore, the combination of a male (MF) and a female (LP) researcher allows for a more nuanced perspective on the nursing sector in Germany, which is predominantly female.

The researchers had no prior personal or professional relationships with any of the participants or the participating institutions before the study commenced. All contact was established solely for the purposes of the research project through formal institutional channels.

Access was established ‘top-down’ through institutional management. This administrative entry initially triggered reservations among some participants, as researchers were occasionally perceived as ‘management auditors’. To address this, we conducted extensive, transparent introductory sessions, explicitly emphasising that participation in the study was entirely voluntary and completely independent of managerial approval or evaluation. By clarifying our academic independence, initial scepticism was significantly mitigated in many instances, and several participants began to view the study as an opportunity to voice professional concerns and to discuss potential challenges in German nursing. Following the presentation of the study in team meetings, no individual nursing staff members who were approached for interviews or focus groups declined participation. However, we acknowledge that the initial selection of participating units was facilitated by nursing management, which may have influenced the sample composition.

Regarding researcher positionality, it should also be noted that both researchers present in the field do not identify as People of Colour. Due to their appearance and German-sounding names, they are ‘read’ as members of the white majority society, regardless of any underlying family migration history. To account for this specific positioning and to mitigate associated ‘blind spots’, the broader project team was intentionally composed of members with diverse cultural and national backgrounds, including People of Colour and researchers with personal migration experiences.

To minimise the ‘observer effect’, we adapted our clothing to the respective settings: wearing clinical scrubs (pool clothing) in hospitals to blend into the professional background and civilian clothes in nursing homes to respect the domestic atmosphere. This choice of attire, as well as the researchers’ role as non-intervening observers, was explicitly coordinated and agreed upon with the respective team leaderships prior to the field phase. This ensured that our presence was transparently integrated into the wards’ daily routines without creating functional ambiguity or disrupting established hierarchies.

### Ethical considerations

The study was approved by the Ethics Committee of Charité - Universitätsmedizin Berlin [EA1/260/21], and the data protection concept was approved by the university’s data protection officer. Participation in the study was voluntary and based on comprehensive advance information. Participation was entirely independent of managerial approval, and nursing staff could decline or withdraw at any time without any consequences. Oral consent was obtained for participant observation due to the anonymity of the data collected. Written consent was obtained from participants in the interviews and focus groups. The transcripts were pseudonymised for analysis; at the end of the project, the pseudonymisation list was deleted, thereby achieving complete anonymisation of the data.

## Results

The findings of this comparative case study are organised into a typology comprising three distinct team types: *Routine-based Teams*, *Transition-based Teams*, and *Friction-based Teams*. To provide a systematic and transparent overview of the empirical boundaries and internal variations of these types, Table 4 maps the six investigated cases against the two core analytical dimensions: (1) the collective orientation towards socio-cultural and qualification-related diversity, and (2) the degree of mutual support within the team and from the team leadership. This matrix also delineates the intra-type variances that characterise the praxeological pathways within each team spectrum.

**Table 4:** Overview of typological dimensions and case-specific core orientations

Team/ Case	Type	Analytical Dimensions for Type assessment		Illustrative Empirical Indicators
		(1) Collective orientation towards socio-cultural and qualification-related diversity	(2) The degree of mutual support within the team and from the team leadership	
Team A	Routine-based Teams • diversity as a positive and identity-forming characteristic • different skills of team members are valued • differences in origin or qualification are integrated into a shared professional identity and everyday routines • strong identification with the nursing team and overarching team identity	Resource-oriented • deep appreciation of diverse socio-cultural and professional backgrounds • intensive exchange of socio-cultural and professional knowledge	Very high level of mutual support • Support involves all team members equally and is not limited to a subgroup	A nurse talks about “social issues” and says they “did a headcount last week.” Apart from himself, there are only three white cis men in the whole team. Everyone else either has a migrant background, is gay or is female. That is why the team is so good; there is a high level of awareness of diversity. […] He also says that the management is very good and always approachable when it comes to problems within the team. (A-PO-3)
Team B		Resource-oriented • Normalisation of different socio-cultural backgrounds • Ongoing processes of negotiation regarding educational background, pathways to qualifications and the division of tasks	High level of mutual support • Support involves all team members equally and is not limited to a subgroup	Nurse 11: “Mrs XY has had eggs …” Doctor: “What has she had?” Nurse 11: “Eggs…what’s that called in German? Egg, egg, egg…egg cells, desire to have children.” They consider what she has now received and how exactly to describe it. No one has the correct term to hand straight away. They then find a correct term they can agree on (I suspect:) egg retrieval. Nurse 11: “Did I say ‘egg’?” (grin) Nurse 7: “Cluck, cluck, cluck.” (mimicking a chicken) General laughter. (B-PO-6)
Team F	Transition-based Teams • Active engagement with the challenges arising from high levels of socio-cultural and educational diversity • diversity as a shared learning task • team integration process encompasses shifting staff configurations	Resource-oriented (practice-aligned) • (Highly) reflective engagement with diversity • Coping with educational diversity leads to changes in organisational structures	High level of mutual support • strong mutual support of new international nurses during integration process	[Meeting between the deputy director and the new ward manager] Deputy director: “Tip … pick some who’re good with medication … you’ve got [Asian first name] and [Asian first name] … they’re quick, they’re good.” It really is a lot of work to do all that; she herself would then only need to carry out spot checks. (F-PO-6)
Team D		Rather resource-oriented (cognitive-bound) • Diversity is recognised and discussed, yet day-to-day practice oscillates between learning and a burden • new integration routines have not yet become established	Moderate level of mutual support: • willingness to actively support international nurses, but hindered by long-standing working practices	For example, I have [a caregiver], who’s still relatively new here, […] he’s from Vietnam, so naturally he still faces a significant language barrier; he’s just a very quiet sort. He doesn’t exactly stand out, does he? But he does a great job. […] And a lot of people just don’t have the patience when he doesn’t know everything yet. And then he gets snappy replies when he does ask something. That happened the other day, and he told me that sometimes he doesn’t even dare to ask anymore. That’s difficult. So I always try to say to the staff: ‘Come on, you’ve got to be patient.’ […] My plan is to keep him on. [laughs] (D-SI-1)
Team C	Friction-based Teams • Low level of active commitment to diversity; a generally negative attitude towards working with international colleagues • Diversity is seen as a disruptive factor • The expertise and qualifications of international nursing staff are called into question • The formation of sub-groups between the core team and international nursing staff undermines team cohesion	Problem-oriented • International carers are perceived as a significant systemic burden and a threat to workplace stability • Emphasis on fundamental conflicts of professional values between German and international carers • Mistrust regarding the qualifications of international carers	Support is kept to the essentials • Support for international nurses is seen a necessity, but it is provided rather reluctantly, with the focus on extra work and additional burdens	The project with “the Vietnamese”, on the other hand, was a “complete failure”. Both [nurses] report this. There were problems here with both language skills and technical work. It later emerged that some of them had bought their qualifications. It was clear that they had never even held a pill in their hands, let alone administered an infusion. Pills are the same all over the world, whatever their specific names may be. But it was obvious that they had never had anything to do with them before. (C-PO-2)
Team E		Problem-oriented • International carers are perceived as a significant systemic burden and a threat to workplace stability • Emphasis on fundamental conflicts of professional values between German and international carers • Mistrust regarding the qualifications of international carers	Support is kept to the essentials • Support for international nurses is seen a necessity, but it is provided rather reluctantly, with the focus on extra work and additional burdens	I strike up a conversation with a nurse who is tidying up the kitchen. […] She is critical of foreign care workers, particularly Muslims. A colleague once said to her: “You’ve got nothing to say to me; women are only here for sex.” She explains: “I’ve got a migrant background myself; they should support those who grew up here first, before bringing in others.” (E-PO-5)

It is essential to clarify that these types are conceptualised as heuristic real types. Rather than representing a normative ranking or a fixed, linear progression of diversity management, they serve as analytical frameworks to map the complex and often non-linear dynamics of team integration.

### The routine-based teams

The Routine-based Teams (A and B) are characterised by high diversity and a conscious awareness of this diversity. For these teams, the existing socio-cultural and qualification-related diversity is explicitly framed as a positive and identity-forming characteristic, rather than as a source of problems. Team members show a strong identification with the nursing team itself as a superordinate group, and differences in origin or qualification are integrated into a shared professional identity and everyday routines.

#### Team A: Valuing diversity as an asset

Team A (hospital) experienced a structural reorganisation through the merger of two distinct wards approximately one year prior to data collection. While participants explicitly mention work-related conflicts during the initial phase of team integration (A-PO-7; A-FG-1; A-SI-4), they retrospectively describe the merger as successful and emphasise the value of varied professional experiences (A-PO-2; A-SI-2; A-SI-3; A-SI-4). Furthermore, the mutual support within the team is repeatedly highlighted (A-SI-2; A-FG-1; A-FG-2), something that was also evident in day-to-day working life (A-PO-2; A-PO-3; A-PO-4; A-PO-5). In Team A, diversity is actively valued as a crucial asset that enhances team performance and fosters a deep sense of belonging (A-PO-2; A-PO-5; A-PO-10, A-SI-5). This appreciative stance toward a diverse team composition is explicitly illustrated in a focus group discussion:

I think it’s great when the team is, um, well mixed in terms of experience. In other words, you have colleagues who have many years of professional experience as well as colleagues who are perhaps still relatively newly qualified, but also colleagues who have already gained experience in different areas and who are a colourful mix. (A-FG-2)

Furthermore, rather than treating diverse backgrounds as a standardising challenge, a nurse explicitly argues in an interview for a personalised and context-specific transfer of knowledge:

Just because I consider things to be self-evident, [laughs] I can’t assume that everyone else considers them self-evident as well. I believe, due to this cultural diversity and the diversity of training and different qualifications that one has […] that one simply shouldn’t lump all these different degrees with different origins from different countries together, but rather one should be a bit more specific. [This] might be helpful for one or the other that it is done the way we envision it here on the ward. (A-SI-5)

Interpretively, the reconstruction of these sequences reveals a shared orientation pattern where socio-cultural and qualification-related diversity is praxeologically evaluated not as a deficit, but as an operational asset. The collective ‘space of experience’ in Team A is characterised by the implicit assumption that varying professional biographies and socio-cultural origins represent an inherent added value that fosters team performance.

Diversity awareness in Team A is not limited to professional qualifications but is linked to societal discourses on diversity. One team member emphasised that the high personal diversity (e.g., migration background) leads to a “high level of sensitivity towards diversity” (A-PO-3), as many are personally affected by or informed about discrimination. This shared sensitivity is understood as a resource for team cohesion.

Framed through the theoretical lens of Social Identity Theory (SIT), these reconstructed patterns demonstrate a process of recategorisation. The primary, decisive in-group for the actors is the superordinate, unified nursing team identity. In other words, in Team A, the formal organisational team is fully congruent with the subjectively perceived team.

By anchoring the shared identity on this higher level, the distinct professional and socio-cultural sub-identities are successfully integrated rather than being perceived as threatening out-groups. Consequently, potential intergroup conflicts are minimised. Although minor intra-group frictions can still be reconstructed – primarily along generational lines regarding working paces (e.g., A-SI-2; A-SI-4; A-PO-3) – the identification with the overarching team identity remains exceedingly dominant. This structural alignment of the ‘administrative ward’ and the subjective ‘felt team’ culminates in a profound sense of belonging, epitomised by the statement: “We know that we stand and fall together” (A-SI-2).

#### Team B: Celebrating cultural mix, negotiating qualifications

Team B (hospital) experienced an organisational restructuring shortly before data collection, which reduced its area of responsibility from two wards to one due to a shortage of qualified personnel. However, because the core team constellation itself remained unchanged, participants did not perceive this structural contraction as a disruptive crisis. Externally, Team B presents itself as a cohesive unit that explicitly celebrates its socio-cultural heterogeneity (B-SI-1; B-SI-2; B-SI-3), which encompasses 14 different languages. Here, too, the participants highlight the support within the team (B-SI-1, B-SI-3; B-SI-4), which was confirmed by observation (B-PO-2; B-PO-7). Daily communication barriers or minor misunderstandings are routinely and collectively defused through humor (B-PO-2; B-PO-3; B-PO-6). This pronounced, positive collective self-perception of being a unique, highly inclusive, and versatile group is vividly illustrated in a focus group discussion:

Bw: I have my team. (.) So I like this, this, such a mix is just amazing. Yeah. Not everyone has that. @(.)@ I like my team. We have the calm ones, the balanced ones, we have the crazy ones, we have the complete idiots, we, @(.)@ we have everything. I think it’s great. We have multiculturalism, we have a lot of humour, we have witches, we have sneaky ones, yeah, it’s good like that. @(.)@ […].

Aw: Yes, we have everything.

Cw: We have everything. @(.)@ (B-FG-3).

However, the data reveals that this celebrated inclusion of diverse personal characteristics masks a severe internal tension regarding professional nursing roles and values. This conflict emerges openly during the focus group: one nurse (Bw) fiercely advocates for an efficiency-driven, functional division of labour, arguing that highly skilled professionals should exclusively perform qualified medical-nursing tasks while delegating basic care to assistants. Conversely, her colleague (Aw) defends a traditional, holistic nursing model, viewing basic care as intrinsically linked to professional identity (B-FG-3). This central negotiation is highly contextualised within current macro-level debates in German nursing, which are shaped by the dual pressures of academicisation, increasing specialisation, and the shifting boundaries between qualified and non-qualified care.

Interpretively, the reconstruction of Team B’s collective ‘space of experience’ exposes a multi-layered orientation pattern. On the first layer – socio-cultural origin – the team has established a highly integrated, habitual routine where varying migration backgrounds are completely normalised and no longer function as a category of difference. Team B is the only case study where the integration of international nursing staff is never discursively problematised. On the second layer – professional qualification – the team orientation is characterised by an active, ongoing negotiation of professional values. Here, belonging to a specific qualification group or holding a particular professional ethos holds a significantly stronger relevance for the actors’ occupational self-conception than their migration history.

Framed through the theoretical lens of Social Identity Theory (SIT), Team B illustrates a highly complex constellation of in-group dynamics. In contrast to Team A, where formal organisation and the subjectively ‘felt’ team are effortlessly congruent, Team B maintains its superordinate team identity (in-group) through the explicit and humorous celebration of personal and socio-cultural diversity. This robust overarching team identity functions as a protective psychological buffer. Because the identification with the unified team is so dominant, the severe professional conflicts regarding roles and division of labour can be debated openly as intra-group friction. These differences of opinion occur strictly between members of the established in-group and do not lead to the formation of out-groups along qualification lines. The overarching team identity remains paramount, allowing the team to absorb deep professional dissent without fracturing its social cohesion.

#### Intra-type heterogeneity within routine-based teams

Within the Routine-based Teams (A and B), both units operate within a structurally stable everyday framework and share a highly appreciative evaluation of their internal diversity. However, their internal dynamics reveal a distinct structural divergence. In Team A, the formal organisational structure and the subjectively felt team are effortlessly congruent, allowing the unit to seamlessly absorb sub-identities into a superordinate team identity. Conversely, Team B’s stability is characterised by an active, ongoing negotiation of professional values. While they successfully normalise and decouple socio-cultural origins from their shared identity, they simultaneously grapple with deep internal tensions regarding qualification lines and task division, transforming their routine into a continuous space of occupational negotiation.

### The transition-based teams

The Transition-based Teams (F and D) are characterised by a high level of awareness of socio-cultural and skill diversity and by an active engagement with the challenges that arise. These teams explicitly reflect on structural barriers and everyday frictions, demonstrating a clear potential for recategorisation and change. While ambivalent attitudes towards working with international nursing professionals can certainly be found, a largely positive social identity is created at the team level that includes (almost) everyone, and prejudices and stereotypes are actively questioned and reduced.

#### Team F: Proactive integration and mutual learning

Team F (nursing home) operates within a newly opened facility, structurally lacking a pre-existing, historically grown “core team” (F-PO-1). Consequently, collaboration between German- and foreign-trained personnel was established as the default institutional setting from the outset, with approximately 20% of the team members being foreign-trained professionals currently navigating the official recognition process (F-SI-1). What is striking about this team is their willingness to break down language barriers during their work by having the experienced carers explain complex issues to their international colleagues in a clear and patient manner, and by regularly checking whether everyone is able to follow (F-PO-1; F-PO-2; F-PO-3; F-PO-7; F-SI-3). International carers are trained in the documentation programme on the job by leading staff, experienced colleagues, and their peers (F-PO-3; F-PO-5; F-PO). Training sessions are also held whenever a particular issue becomes a hot topic (F-PO-2; F-PO-5; F-PO-6; F-SI-1). Moreover, this team proactively manages diversity by implementing structured, practice-oriented integration measures based on mentorship, as one leading nurse states in an interview (F-SI-1). In a focus group, too, an experienced nurse highlights this point:

Those we have on our ward now, it was very difficult at the beginning because communication was practically non-existent. […] things have settled down very well now because our skilled staff, I’d say, the ones who were already here, they’re really taking them under their wing, so to speak, and showing them a lot. (F-FG-7)

Crucially, this knowledge transfer is not restricted to formal nursing hierarchies; assistant staff also actively pass on practical expertise regarding daily basic care routines to foreign-trained colleagues:

It was hard for [our foreign-trained colleague], but we also try to get them to join our team. We really are a great team and we really take them with us and draw them along with us. I’m just a pleb, I always say, I’m just a pleb because I’m just a carer, yeah? But I can teach them a lot, because I’ve been in caring for a few [years] and they’re grateful for that, because I know how to wash people or stuff like that. They just didn’t know that you do intimate care in the evening or that you brush your teeth in the evening. They didn’t know any of that. It’s not their fault! They didn’t learn any differently. (F-FG-7)

This significant additional everyday effort required for mutual integration is explicitly accepted by the staff, who frequently refer to themselves as a “dream team” (F-FG-7). This high investment in collaborative growth is further illustrated by an established international nurse who continues the cycle of mentorship:

I just know that I have colleagues that I can count on, that’s enough for me. […] If you have easy work later on, then why shouldn’t you work a bit on your colleague’s form? (F-FG-7)

Interpretively, the reconstruction of Team F’s collective ‘space of experience’ reveals a dynamic, transition-oriented pattern focused on resource-oriented inclusion. The team’s habitual practice is characterised by a high willingness to blur traditional boundaries of origin and hierarchy through bi-directional learning. The knowledge gaps of international colleagues are empathetically reconstructed not as individual incompetence or professional deficits, but as structural differences in international curricula. This perspective prevents negative stereotyping. Furthermore, the learning process is reciprocal: while established staff teach local basic care routines, they simultaneously seek to learn from the advanced clinical treatment skills of their new international colleagues (F-PO-6, F-FG-8).

Framed through the theoretical lens of Social Identity Theory (SIT), Team F illustrates a successful, ongoing process of recategorisation through boundary expansion. Initially, the lack of shared norms and severe communication barriers led to a classic in-group/out-group differentiation between the established staff and the newly arrived international newcomers. However, instead of consolidating these frontlines, the team actively reconstructed its in-group boundaries to systematically incorporate the former out-group.

The language used by the actors (“our team”, “get them to join our team”) theoretically signals a strong willingness to broaden the category of belonging. By framing the daily evaluation of skills as a positive, mutual knowledge transfer rather than a basis for structural ‘othering’, the emerging differences are successfully absorbed. Backed by shared organisational goals and a highly resilient, valued team identity (“dream team”), the administrative structure and the subjectively felt team are continuously aligning toward a unified, inclusive in-group, where boundaries are actively expanded to absorb external strains through mutual support.

#### Team D: Intellectual validation and unrealised professional transition

Team D (hospital) is the only investigated unit that has not undergone any recent, disruptive structural reorganisations that could have jeopardised team cohesion. Consequently, Team D is a relatively robust and well-established team that exhibits a high level of cohesion and mutual support (D-PO-7; D-SI-1). However, according to a senior nurse, this also reflects a certain resistance to changes in established working practices (D-SI-1). In regard to the matter of team diversity, participants exhibit a marked ambivalence in their attitudes. On the one hand, there is explicit empathy for new international staff members and acknowledgement of their advanced skills and motivation (D-PO-4; D-SI-1; D-FG-5), on the other hand individual interviews express isolated xenophobic sentiments (D-SI-2/3). This ambivalence and a clear discursive categorisation into two distinct groups – German nursing staff’ and ‘foreign nursing staff’ – is illustrated in a focus group sequence focusing on the mismatch of international training standards:

In principle, as I said, the idea of bringing in skilled workers from abroad is not wrong in itself, but (.) as I said, because of the different training, because the nurses there have a different status in general, it won’t work that way. Because they’ll all come here and say: Am I stupid? I don’t do that here. [laughter] I don’t need that in my country, I have a different status there. Why should it change for them? As I said, I think that’s the main problem and it’s nothing new. I’ve already been with those who came from Mexico and Spain. They all went back and said: ‘Please! I’m not doing this job here for the money! [Murmurs of agreement from the others] (D-FG-5).

The focus group explicitly attributes this structural friction to the perceived devaluation of academic qualifications held by foreign-trained nurses when confronted with the German focus on basic care:

Ew: Basic care is not part of their training. That’s like telling a doctor to throw away your academic degree and go and do the washing! Find that doctor.

Cw: That’s three steps back.

Ew: Yes! That’s why that principle won’t work here. […] Because you’re actually downgrading them. (D-FG-5)

Despite this rigid boundary-drawing, the data highlights a capacity for joint professional reflection when a foreign-trained colleague (Fw) introduces an alternative, academically driven sequence of care (prioritising vital signs over immediate washing):

Fw: Nah, I think here, this situation, um, normally, when you walk into the room, there are two different nurses. One German and one from Mexico … so where we have learnt, first the patients are not allowed to get up, must first have their vital signs taken, then they are washed, taken to the toilet, because if they start to move, the vital signs go up, then it’s not normal. So. That’s why I think @Rosita@ wanted to do this first before they start nursing. […] that’s how we learnt first. First the vital signs, then go to the carer to wash, like this!

Dw: It’s actually the right thing to do. It’s just that we have a different plan in mind, I have a list in my head.

Fw: Don’t wash straight away. First the vital signs, then you catch your … the bowl, you accompany the patients and so and so. […]

Aw: Everyone has their job here!

Fw: Everyone has a task ….

Aw: Exactly! (D-FG-5)

Interpretively, the reconstruction of Team D’s collective ‘space of experience’ reveals a highly complex, reflective, yet practically stagnant orientation pattern. The core interpretive pattern is characterised by intellectual validation coupled with habitual inertia: the team explicitly acknowledges the validity and superiority of diverse, internationally acquired professional knowledge (“It’s actually the right thing to do”), yet they explicitly state an inability to adapt their own deeply ingrained, internalised daily work routines (“I have a list in my head”). Learning is thus decoupled; it occurs as a shared cognitive reflection but does not manifest as an altered praxeological routine. Moreover, when analysing a negative integration experience with a foreign nurse a few month ago during the focus group discussion, the team collectively abstracts the event, interpreting it as an “isolated incident” or structural “bad luck” caused by inflexible corporate staffing (D-FG-5).

Framed through the theoretical lens of Social Identity Theory (SIT), Team D demonstrates the rigid limits of recategorisation within a structurally flawed environment. The primary in-group (German staff) and out-group (foreign staff) boundaries are highly consolidated along the dimensions of training background and professional status. However, the associated in-group bias operates in a paradoxical manner: instead of devaluing the out-group to elevate the self, the speakers perform a social comparison that portrays the out-group as structurally privileged and unjustifiably downgraded by the German system. The phrase “Am I stupid? I don’t do that here” functions as a strong, reflected devaluation of their own, German care system rather than an attack on the newcomers.

While the shared professional reflection around clinical sequences signals a momentary, burgeoning potential for recategorisation and mutual learning, the structural alignment between the formal organisational framework and the subjectively felt team remains blocked. The dominant sub-identities do not merge because the established in-group members ultimately prioritise their defensive, internalised “mental lists” over inclusive practice. Team D thus marks a specific transitional space where systemic empathy is high, but inclusive operational change remains unrealised.

#### Intra-type heterogeneity within transition-based teams

While both Transition‑based Teams (D and F) share a highly reflective engagement with institutional diversity, they exhibit crucial differences in their praxeological translation. Team F displays a proactive, dynamic boundary expansion within a newly established facility, successfully shifting the formal administrative unit and the subjectively felt team into alignment. Conversely, Team D’s transitional trajectory remains largely cognitive; their reflection is characterised by high systemic empathy, yet the actual adaptation of practices remains unrealised, heavily restricted by long-standing, defensive operational routines.

### The friction-based teams

The Friction-based Teams (C and E) are highly diverse in composition but display a low sensitivity to diversity. While socio-cultural and educational differences are clearly present, there is little active engagement with them; instead, the knowledge and qualifications of international colleagues are frequently questioned. As a result, subgroup formations along social categories become more likely, which in turn fosters conflict and undermines cohesion at the team level.

#### Team C: Differentiation and avoidance of conflict

Team C (hospital) is an established, structurally stable unit characterised by a high proportion of long-term employees and dense, well-developed social ties (C-PO-3; C-SI-4). These ties include international staff members who report feeling accepted (C-SI-2). This configuration has fostered a pronounced resistance to changes in work processes (C-SI-1; C-FG-4). While the team recently received a new ward manager who is evaluated positively by the staff (C-SI-2; C-SI-4), the arrival of international colleagues is discursively framed as a profound systemic burden and a threat to workplace stability.

This defensive positioning is illustrated in a focus group sequence where a senior nurse outlines a fundamental clash of professional values, explicitly contrasting the German system with international training backgrounds:

In many countries, care plays a less significant role because it is mostly provided by family members. This is handled quite differently in Germany. […] However, here in Germany, the focus is on care, which sometimes leads to clashes. There are some who come from abroad, outside of Germany, and are recognised here, and it becomes clear that there is a much stronger emphasis placed on the medical aspects compared to care. (C-FG-4)

This professional differentiation rapidly scales up into explicit suspicion regarding the professional competence and moral integrity of the incoming international colleagues. In an individual interview, a nurse frames the arrival of multiple international newcomers as an unmanageable challenge, culminating in accusations of document fraud:

Well, that’s a completely different education, and they are not familiar with things here at all. […] And if four of them come at once, and we are supposed to integrate them into our work processes here, then that’s already difficult. That is really difficult. It’s not a question of not wanting to or not being willing to, but it’s simply difficult. […] I really know this from my old facility: They come and think they can do everything, but there’s not much behind it. So I also have to be careful that they haven’t bought their degrees somewhere. Not so easy. (C-SI-1)

Interpretively, the reconstruction of Team C’s collective ‘space of experience’ reveals a rigid, defensive orientation pattern rooted in a deficit-oriented view of alterity. The collective habitus relies on a severe binary opposition: “German nursing” is implicitly constructed as a superior, holistic practice, whereas “international nursing” is reduced to a purely technical, medical orientation that lacks essential caring values.

A striking argumentative shift becomes visible here: while the integration challenges are initially framed as objective, organisational hurdles (‘simply difficult’), the discourse progressively transforms during the interaction into a moral stigmatisation. Through this collectively shared suspicion regarding the authenticity of foreign educational degrees, the team preemptively strips the newcomers of any professional legitimacy. This refers in particular to a previous project with “the Vietnamese”, which was a “complete failure” (C-PO-2) and left the team feeling deeply frustrated. Consequently, differences are not understood as a starting point for negotiation or mutual learning but are instead reenacted as insurmountable deficits and lead to a more cautious approach to future recruitment projects (C-PO-2; C-SI-1; C-SI-2).

Framed through the theoretical lens of Social Identity Theory (SIT), Team C demonstrates a classic, polarising manifestation of in-group favoritism and hostile out-group derogation. The deeply entrenched, established team functions as a rigid in-group that protects its status quo by stereotyping the international out-group. To justify an ‘us versus them’ mentality, the actors utilise the dimensions of qualification and nursing philosophy as non-negotiable exclusionary boundary markers.

This specific configuration highlights clear structural contrasts within the typology: Unlike the stability-based ambivalence in transition-based Team D – where routines are maintained but the systemic flaws are intellectually reflected – Team C exhibits a non-abstract, personalised resistance to change. Furthermore, Team C’s exclusionary use of professional backgrounds contrasts sharply with routine-based Team B, where diverse professional identities are negotiated openly as a shared resource, successfully decoupling professional competence from socio-cultural origin. In Team C, a deep structural chasm emerges between the formal administrative unit and the subjectively felt team: while the international newcomers are institutionally already part of the administrative team, the established staff close themselves off completely, rejecting their integration to protect the historical boundaries of the in-group.

#### Team E: Cultural clashes and unmanaged multi-group dynamics

Team E (nursing home) operates under the persistent strain of multiple prior structural crises, which have profoundly weakened team cohesion and created deep internal divisions. These crises – namely a chaotic change of facility ownership and the severe institutional impact of the COVID-19 pandemic (E-PO-1; E-PO-2; E-PO-3) – fostered highly polarising group dynamics. The shared trauma of chronic overwork and massive staff turnover during the pandemic created a fertile ground for social comparison and prejudice.

This intersection of ethnic identity and professional status becomes evident when staff members discuss colleagues leaving the facility, explicitly labeling those who exit as opportunistic and those who remain as heroic: one nurse states that “the Vietnamese tend to go to the hospitals, to the intensive care units, where they don’t have to wash” (E-PO-2). Furthermore, a focus group session involving both German and established foreign-trained nurses demonstrates a fierce, defensive hostility toward newly arriving international professionals:

Bw: We’re getting […] two new Turkish (.) people, which we’re happy about, but when I hear they’ve just arrived, it makes my hair stand on end [gestures her hair standing on end]. Honestly, I’m glad I have three weeks of vacation [Bw giggles, everyone laughs] […] look at [the colleague from Turkey]. […] She has no idea where she’s landed. Does she even want to do this? She’s doing it so she can stay in Germany. […] are they even really trained, honestly [Aw], have you seen any evidence of their prior qualifications? No. (E-FG-6)

This structural fragmentation is explicitly confirmed by the participants themselves. One established international nurse explicitly decouples mere technical cooperation from a shared social identity: “We don’t have a team. We just work together. […] There are two or three people here who like to work together. And the others. But we’re not a team” (E-PO-2). This diagnostic breakdown is corroborated by widespread reports of dysfunctional or entirely absent communication (E-SI-1; E-SI-2; E-SI-3; E-FG-6) and has also been observed on several occasions (E-PO-2; E-PO-4; E-PO-10). The data also reveals xenophobic comments (E-PO-5; E-PO-7; E-FG-6). At the same time, an international care worker explains that she encounters fewer prejudices here than at her previous workplace (E-SI-I).

Interpretively, the reconstruction of Team E’s collective ‘space of experience’ reveals a habitus of chronic exhaustion and a completely deficit-oriented framework of interpretation. The arrival of international colleagues is praxeologically filtered not as an organisational resource, but strictly as an impending physical threat and an unmanageable professional burden. The metaphor of the “hair standing on end” and the collective laughter about “fleeing” into vacation document a collective coping mechanism rooted in structural fatigue.

Crucially, the aggressive questioning of the newcomers’ actual qualification certificates serves as a psychological defense mechanism. It allows the established staff to preemptively rationalise their rejection of the newcomers, projecting the looming ‘hidden work’ of constant supervision and potential overload onto the alleged incompetence or instrumental motives (“so she can stay in Germany”) of the international colleagues.

Framed through the theoretical lens of Social Identity Theory (SIT), Team E represents a state of acute social fragmentation where a superordinate in-group identity has entirely ceased to exist. The institutional shocks of the multi-crisis environment have completely eroded the overarching team category. Instead of a unified nursing team, the social unit has fractured into localised, unstable sub-groups (“two or three people… and the others”).

Consequently, there is rarely psychological buffer or shared purpose left to absorb organisational strain. A profound structural chasm separates the formal administrative unit from the social reality: while the clinic management continuously adds new international staff to the formal roster, the subjectively felt team consists only of isolated factions. In this dysfunctional environment, ethnic and professional differences are weaponised as rigid, non-negotiable markers of exclusion to keep the crumbling boundaries of the remaining sub-groups intact.

#### Intra-type heterogeneity within friction-based Teams

The Friction‑based Teams (C and E) both display deeply defensive, exclusionary orientations, yet their structural pathways diverge significantly. Team E’s rejection of diversity is reactively driven by a traumatic multi-crisis history (ownership changes, the pandemic, and high staff turnover), resulting in a fragmented social unit and a habitus of utter exhaustion. In contrast, Team C’s ‘us versus them’ dichotomy operates within a highly stable, well-integrated everyday environment, where exclusion is utilised actively and structurally to protect traditional vocational values and historical in-group boundaries against a perceived external threat.

### Factors influencing how diversity is managed within care teams

Having reconstructed three distinct types of nursing teams in their approach to socio-cultural and educational diversity, the analysis turns to the structural configurations and contextual patterns associated with the emergence of these collective orientations. Across the sample, the two Friction‑based Teams (C and E) and the two Transition‑based Teams (D and F) are distributed across both acute care hospitals and long-term nursing homes, whereas the two Routine‑based Teams (A and B) operate within the same hospital. This empirical distribution demonstrates that, contrary to initial assumptions, the broad institutional macro-setting (hospital vs. nursing home) alone does not systematically correspond with the three diversity types. Consequently, while the qualitative nature and the limited sample size of six cases preclude any direct causal claims, a systematic cross-case comparison highlights two relational dimensions as particularly salient for shaping team dynamics: the socio‑cultural environment of the facility – including its specific patient population – and the localised leadership practices within the teams. Table 5 presents an overview of these factors among the team types.

**Table 5:** Overview of factors that influence dealing with diversity in nursing teams

Team Type	Socio-cultural environment and patienthood	The role of leadership
Routine-based Teams	Multicultural inner-city hospital with a highly diverse patient population and long-standing local discourses on socio-cultural diversity. Impact on team dynamics: The socio-culturally diverse patient population means that the linguistic and socio-cultural diversity of the nursing staff is valued in patient care	Managers embed integration in core routines through standardised induction and expert groups or high-trust delegation and self-scheduling. Impact on team dynamics: A structured and facilitative leadership style (Team A) or one based on a high level of trust (Team B) enables the teams to proactively address socio-cultural and educational diversity in their day-to-day care.
Transition-based Teams	Comparatively more socio-culturally homogeneous environment and patienthood. Impact on team dynamics: As there is little external pressure to provide culturally sensitive care, the focus is on negotiation processes and integration measures with regard to teamwork.	Managers implement mentoring structures, act as advocates for international nurses and proactively mediate conflicts. Impact on team dynamics: Supportive leadership provides the team with a vital framework for navigating diversity-related changes – even if the practical measures taken vary from case to case.
Friction-based Teams	Comparatively more socio-culturally homogeneous environment and patienthood. Impact on team dynamics: As there is little external pressure to provide culturally sensitive care, the focus is on negotiation processes and integration measures with regard to teamwork.	Managers share deficit-oriented views of international staff, mistrust recognition procedures and intervene mainly reactively. Impact on team dynamics: Due to negative experiences in the past, when they felt inadequately prepared for the integration of international carers, these managers tend to adopt a largely reactive stance. Integration is rarely promoted proactively, partly because of the teams’ sceptical or even resistant attitude.

#### Socio-cultural environment and patienthood

At the descriptive level, a systematic cross-case comparison reveals that the broad institutional setting (hospital vs. nursing home) alone does not systematically map onto the three team types. Instead, the specific socio-cultural environment and the patient population of the respective facility show a more consistent association with the teams’ diversity orientations.

The Routine-based Teams (A and B) operate in a hospital located in a urban neighbourhood with a historically established, highly visible migrant population – a social space locally perceived and discursively celebrated as “multicultural” for decades. A key empirical characteristic of these teams is their explicit, continuous discourse regarding intercultural patient care. Staff members frequently reflect on how the diverse linguistic and socio-cultural backgrounds of international colleagues provide a direct professional benefit for treating a diverse clientele. Employees regularly utilise their skills for language mediation, not only providing care on their own ward (A-PO-4; A-PO-5; B-PO-3; B-PO-6) but also acting as central “contact persons” for other hospital departments (B-SI-3).

Furthermore, the data documents a highly developed awareness of how communication barriers structurally affect care delivery. For instance, a ward manager explicitly addresses during handover that a patient’s perceived “non-compliance” is rooted in a language barrier rather than individual resistance (A-PO-2), noting that communication in the patient’s native language is a key clinical resource “because it simply defuses situations quite often” (A-SI-5). This knowledge transfer also encompasses specific health concepts; leaders emphasise that “it’s incredibly valuable to have someone” who can explain culturally specific understandings of illness (TL-KH1-A-2). This sensitivity extends to gender-sensitive care preferences (A-SI-3) and the pragmatic integration of family routines, such as accommodating home-cooked food during pandemic restrictions (A-PO-6).

By contrast, the Transition-based (D and F) and Friction-based Teams (C and E) operate in comparatively more socio-culturally homogeneous environments. A striking empirical characteristic across these four cases is the near-total absence of an explicit discourse on intercultural patient care. The cultural and linguistic resources of international colleagues remain largely invisible in daily clinical routines. In these settings, diversity appears almost exclusively in narratives regarding internal coordination, communication barriers, and individual workload, rather than in connection with patient care benefits.

Interpretively, the reconstruction of these patterns highlights how the socio-cultural environment functions as a continuous background condition that provides specific interactional resources. In the Routine-based teams, everyday exposure to diversity – both in private spheres and in professional contact with a diverse patient base – allows socio-cultural difference to be experienced as an everyday normality rather than an exceptional organisational disruption. Because the linguistic and cultural skills of international staff are routinely required to achieve daily work goals, their ‘otherness’ is pragmatically reframed as an operational asset. Conversely, in the Transition- and Friction-based teams, the lack of externally anchored, diversity-related demands means that diversity is rarely experienced as a functional resource for the primary care task. Instead, it is interpreted primarily as an internal organisational challenge.

Framed through the theoretical lens of Social Identity Theory (SIT), the empirical link between the environment and team dynamics can be conceptualised through the mechanism of functional anchoring. The external diversity of the neighbourhood and patient population functions as a structural anchor that facilitates internal cohesion. By explicitly tying the team’s shared professional mission to the demands of a diverse environment, the superordinate in-group identity is constructed to inherently include diversity.

Crucially, this external anchoring prevents the formation of hostile out-groups along national lines, because the specific skills of international colleagues are vital for the in-group’s collective success. In teams where this external anchor is missing (the Transition- and Friction-based cases), the team baseline remains monocultural. In these contexts, the data suggests that the teams’ developmental trajectories – whether they move toward inclusive transition or polarising friction – cannot only rely on external environmental incentives but seem to depend more on internal managerial boundary work.

#### The role of leadership

The comparative cross-case analysis indicates that leadership – comprising both ward managers and their deputies – acts as a central mechanism in shaping how teams respond to diversity. While the socio-cultural environment establishes a contextual baseline, specific leadership practices are closely associated with whether a team develops inclusive (Routine- or Transition-based) or exclusionary (Friction-based) patterns. Empirically, these practices manifest across three distinct dimensions: the structural design of induction processes, everyday boundary-setting around professional competence, and active mediation between established and international staff.

In both Routine-based Teams (A and B), managers embed integration into core operational routines rather than treating it as a separate project. However, a cross-case differentiation reveals distinct internal strategies: In Team A, the implementation of a highly standardised induction process for all new employees (A-FG-1; A-SI-2) combined with the establishment of clinical expert groups (A-SI-4, A-PO-3) renders access to knowledge and institutional responsibilities transparent. This signals to the team that qualification and competence, rather than socio-cultural origin, is the sole relevant criterion for professional recognition. The team particularly highlights the management’s proactive improvements, noting that they “communicate, make decisions and get things done” (A-PO-3). Conversely, in Team B, a high-trust, supportive, and delegated leadership style (B-SI-1; B-SI-2; B-SI-3; B-SI-4) – exemplified by family-friendly working hours through staggered shifts and self-scheduling practices – fosters a decentralised team structure where socio-cultural backgrounds remain organisationally neutral in daily practice.

The role of nursing managers becomes particularly transparent when observing how change processes are handled within the Transition-based and Friction-based Teams, where an environmental ‘tailwind’ of external diversity is absent.

In the Transition-based Teams (D and F), leaders adopt a proactive role to bridge the experience gap between established and newly arrived nurses, attaching explicit value to team development and framing the influx of international staff as a positive impulse for organisational change (F-SI-1; F-PO-1). However, a deeper cross-case contrast reveals a substantial difference in the intensity and structural execution of this support. In Team F, the leadership operates with a significantly higher level of active, systematic intervention; the management team establishes highly visible supportive structures, acts continuously as institutional advocates, and actively moderates daily integration processes to shield international nurses from systemic pressure (F-PO-2; F-SI-1; F-PO-6). In Team D, by comparison, while the manager displays a strong willingness to support newcomers and actively appeals to the staff’s patience (D-SI-1), this proactive stance remains more individualised and is hindered or slowed down by long-standing ward practices, resulting in a more moderate level of operational execution. Empirically, despite these varying degrees of activity, both managers prioritise stable team dynamics over minor process-related frictions (D-SI-1; F-SI-1; F-SI-2; F-PO-6) and intentionally raise the team’s awareness regarding the specific integration needs of newcomers through visible commitment.

By contrast, the ward managers in the Friction-based Teams (C and E) display a uniformly sceptic and reactive leadership style, which stands in sharp contrast to the stark structural differences between the two units themselves: while Team C represents a established and cohesive core team, Team E is a fractured unit divided by ongoing internal crises. Despite these divergent team baselines, the managers in both cases articulate a similar, distinct deficit-oriented view that devalues the professional and linguistic skills of newcomers (C-SI-1; E-SI-3) and voices substantial mistrust toward official recognition procedures of foreign degrees (C-SI-1; E-FG-6). Driven by negative past experiences of feeling underprepared for staffing changes, these leaders adopt a predominantly reactive stance. Consequently, the integration process is rarely moderated proactively, and structured strategies to address high turnover rates remain rather absent from their everyday management repertoire (C-SI-1; E-SI-3).

Interpretively, the reconstruction of these leadership styles highlights how management staff provide the primary interpretive frames that teams utilise to make sense of diversity. In the Routine- and Transition-based teams, managerial practices actively model a frame in which socio-cultural differences are associated with shared learning, structural transparency, and mutual professional growth. In sharp contrast, the leadership practices in the Friction-based teams reinforce a deficit-oriented interpretive frame. By mirroring and validating the team’s anxieties regarding workload and the lack of competence of international nurses, these managers provide a rationale that legitimises the team’s resistance to change. Their reactive stance allows existing institutional reservations to persist unaddressed and effectively confirms exclusionary everyday routines.

Framed through the theoretical lens of Social Identity Theory (SIT), leadership operates as the primary mechanism for institutional identity framing and boundary work. Leaders possess the structural power to define the criteria that constitute the team’s in-group. In the Routine-based teams, managerial practices stabilise a superordinate professional in-group based on transparent competence (Team A) or shared operational responsibility (Team B), effectively reducing the psychological opportunities for everyday othering. In the Transition-based teams, managers actively perform inclusive boundary work; by serving as structural mediators and explicitly valuing international expertise, they transform diversity into a shared learning task, thereby systematically expanding the in-group boundaries to absorb newcomers. In the Friction-based teams, however, leaders inadvertently drive in-group favouritism and out-group derogation. By publicly questioning qualifications and focusing on linguistic deficits, they weaponise professional status as a rigid, exclusionary boundary marker. Consequently, the formal administrative unit and the subjectively felt team fall apart, as the leaders’ own defensive framing blocks the recategorisation processes necessary to form a unified social unit.

## Discussion

The empirical reconstruction of the six cases reveals three distinct configurations of how nursing teams navigate socio-cultural and educational diversity, shedding light on the complex interplay between localised practices and broader institutional conditions. Routine-based Teams integrate diversity into stable everyday routines by anchoring it as an operational asset. Transition-based Teams demonstrate high awareness and actively reflect on structural boundaries, though Team D struggles to formalise these efforts. Friction-based Teams, despite high diversity, fall back on defensive, exclusionary routines. Crucially, the primary differentiator between these configurations lies not in the presence or absence of integration challenges, but in the interpretive frames and organisational resources available to address them.

Our findings indicate that the geographical location and the demographic patient mix function as significant, non-causal background conditions. Facilities embedded in historically multicultural neighbourhoods (Teams A and B) systematically link staff diversity to patient care benefits. Conversely, in socio-culturally homogeneous settings (Teams C, D, E, and F), diversity is framed primarily as an internal organisational challenge. However, a strict empirical cross-case contrast cautions against an environment-deterministic view. The fact that Friction-based and Transition-based Teams coexist within identical macro-settings demonstrates that neighbourhood composition alone cannot explain team outcomes. Instead, alternative structural variables – most notably chronic staffing shortages, fluctuating workloads, and localised organisational histories – seem to be equally powerful explanatory factors.

Extensive research on labour market integration in German healthcare highlights that professional and organisational conflicts are frequently “culturalised” – meaning that structurally rooted frictions are essentialised as unchangeable cultural traits [29]. Our typology significantly refines this undifferentiated picture. The empirical data demonstrates that this essentialising mode is exclusively concentrated within the Friction-based Teams (particularly Team E). By contrast, Routine- and Transition-based Teams evaluate challenges through the lens of differing professional socialisation and divergent international training pathways. Within these inclusive team cultures, international nurses are recognised as present or prospective members of the professional community, mirroring prior evidence that a shared sense of collective coping is vital for sustainable workplace integration [30–33].

Importantly, our findings complement this qualitative pattern with quantitative data from our related mixed-methods vignette survey. The survey indicates that while nurses’ evaluations of competence are indeed tied to diversity characteristics (migration background and qualification), their willingness to cooperate practically (e.g., swapping shifts) depends far more heavily on the length of prior collaboration [34]. This empirical triangulation underscores that hostile team environments are not a universal, unchangeable law of international recruitment, but are deeply contingent upon the specific organisational meso-level structure of the ward.

While language and communication barriers represent a ubiquitous challenge across global nursing migration [7, 10, 14, 35, 36], our comparative analysis highlights stark differences in how these barriers are structurally managed. The Friction-based Teams perceive language barriers as a serious systemic disruption, with communication difficulties leading to disruptions in nursing processes. The Transition-based Teams (particularly Team F) organise language support as a shared task within day-to-day nursing practice. The Routine-based Teams actively use multilingualism as a clinical resource to defuse crises when dealing with a diverse patient group.

To avoid an over-reliance on a purely socio-psychological framework, these interactional patterns must be evaluated alongside a structural alternative: resource exhaustion. The escalation of language barriers into communication crises in Friction-based Teams (C and E) directly coincides with extreme workload and critical staffing deficits. Empirically, these structural pressures leave established staff with virtually zero time or energy for stepwise language mentoring. Consequently, what appears on the surface as an individual deficit or cultural resistance is, under closer analytical scrutiny, a direct consequence of a high-pressure environment that structurally punishes the additional communicative effort required for integration. Our typology thus extends previous literature by illustrating that whether linguistic proximity is activated as an asset or blocked as a burden depends heavily on whether the organisation provides the structural buffer-time necessary to manage it.

A similar divergence emerges regarding the (de)valuation of foreign professional knowledge. In Friction-based Teams, we observed manifest role conflicts where highly qualified international nurses were restricted to basic, low-skill tasks. While Routine- and Transition-based Teams utilise knowledge-sharing to renegotiate professional hierarchies, Friction-based Teams remain trapped in rigid, formal boundaries. This closely aligns with literature linking teamwork preferences to knowledge-sharing behaviors [37]. Once again, our qualitative findings converge with our survey data, which confirmed that implicit evaluation logics systematically link social categories to perceived clinical competence [34]. However, these everyday practices are structurally bounded by external organisational factors, including formal job descriptions, rigid staffing norms, and delayed administrative recognition procedures [23]. Frontline teams often operate within narrow institutional corridors where the flexible deployment of international skills is legally or organisationally restricted, meaning that professional underutilisation is frequently an institutional constraint rather than a purely localised team deficit.

Corroborating existing literature, German healthcare institutions frequently maintain a unilateral expectation of adaptation, demanding that international nurses assimilate into inflexible existing structures while leaving ward teams feeling textually and organisationally abandoned [23, 29, 30, 38]. While our study confirms that this pressure for one-sided adjustment risks reproducing structural asymmetries, our typology reveals major differences in how frontline managers mediate this burden. While inclusive leaders in the Routine- and Transition-based teams actively establish localised support structures, managers in Friction-based teams adopt a predominantly reactive, sceptical stance. This empirical divergence closely resonates with international literature emphasising that diversity-sensitive leadership and the active modeling of psychological safety are pivotal for successful team integration [16, 19, 39, 40].

Crucially, this lack of proactive leadership cannot be attributed to individual manager incompetence or a lack of personal cultural competence. Our data shows that the leader of the Friction-based Teams are leading under particularly difficult conditions. They lead units characterised by historical multi-crises, structural neglect, and a lack of organisational preparation for international recruitment. Research shows, a better teamwork climate is associated with higher perception of organisational cultural competence [41]. Therefore, even if individual willingness to change is present, frontline leaders are heavily constrained if higher-level corporate strategies and corporate cultural competence remain underdeveloped. The defensive framing adopted by these managers must therefore be interpreted as a structural coping mechanism in response to systemic institutional under-resourcing.

### Theoretical reflexivity: scope and limitations of social identity theory (SIT)

Throughout this discussion, Social Identity Theory (SIT) provides a powerful analytical lens to decode the micro-dynamics of team integration, specifically the shifting salience of in-group and out-group categorisations, inclusive boundary work, and recategorisation processes. However, SIT cannot serve as an exhaustive or exclusive explanatory framework for the phenomenon under study. The social construction of ‘we-ness’ on a hospital ward does not occur in a vacuum; it is fundamentally shaped and bounded by material, structural, and institutional realities. Chronic understaffing and excessive workloads are not merely cognitive categories – they are material pressures that dictate whether a team has the cognitive and operational capacity to expand its in-group boundaries.

Therefore, while SIT successfully illuminates how categorisation and polarisation unfold in everyday face-to-face interactions, it cannot explain the structural triggers that set these dynamics in motion. To achieve a comprehensive understanding of diversity management in healthcare, SIT must be fruitfully combined with structural perspectives – such as theories of organisational culture, or institutional change frameworks.

### Limitations

While this study offers deep insights into the micro-dynamics of nursing teams, several methodological limitations warrant consideration: First, the qualitative nature and the sample size of six case studies preclude the formulation of a definitive taxonomy. This inductive classification emerged during the research process rather than being predefined in the initial design. Consequently, the typology must be understood as a heuristic framework that maps distinct socio-cultural configurations rather than a fixed, universally applicable model. Furthermore, the restriction of the sample to urban and metropolitan areas limits the immediate transferability of the findings to rural or small-town healthcare settings, where organisational resource structures and community demographics may differ significantly.

Second, access to the empirical field was structurally mediated through nursing management. This institutional gatekeeping may have subtly shaped the sample composition by favouring wards with comparatively stable team dynamics, proactive leadership, or a higher baseline readiness for external scrutiny. To mitigate this potential selection bias, we emphasised during the recruitment phase that participation was entirely voluntary and independent of managerial approval. Empirically, the fact that our final sample successfully captured highly conflicted and fragmented team environments (the Friction-based Teams) demonstrates that management gatekeeping did not result in a sanitised or overly optimistic selection of cases.

Third, because all focus groups were conducted in German, a subtle language-related participation bias cannot be entirely ruled out. International nurses with lower linguistic confidence might have felt restrained from contributing nuanced arguments in a group setting. We actively sought to mitigate this barrier by adapting our moderation style to plain German and fostering a supportive environment that encouraged spontaneous peer-translation among colleagues. However, because we did not conduct subsequent transcript validation (member checking) with individual participants, the extent to which highly subtle or non-verbal meanings could be verified ex post remains limited.

Fourth, the positionality of the two field researchers – as members of the white majority society and as external academics – introduces potential interpretive blind spots. This structural power asymmetry may have influenced data generation, particularly by shaping how openly or defensively experiences of everyday discrimination, xenophobia, or professional devaluation were articulated by international staff. To structurally counter these biases, we implemented a multi-layered reflexivity strategy: preliminary data and raw transcripts were continuously subjected to analysis within multi-disciplinary interpretation groups, and we held participatory validation workshops where our case interpretations were presented to frontline practitioners for critical feedback and correction.

## Conclusion

Nursing teams develop diverse strategies for managing cultural and skill diversity. To foster a diversity-sensitive culture, proactive support from all management levels is essential, particularly by providing time and space for mutual exchange. Managers should implement targeted team-building activities and structural interventions – such as supervision or improved physical environments (e.g., team rooms) – to strengthen shared identity. Measures should be tailored to specific team types:

- *Routine-based Teams* benefit from brief inputs on international nursing contexts during meetings.
- *Transition-based Teams* require structural support for collaborative learning and informal exchange, such as regular team breakfasts.
- *Friction-based Teams* necessitate comprehensive organisational development, including cultural competence training, change management, and leadership involvement.

Diversity itself is not the hurdle; rather, the organisational context dictates its impact. By consciously designing work environments, organisations can transform diversity into a productive asset. Future longitudinal research should evaluate the long-term impact of these interventions, using the identified typology – routine-based, friction-based, and transition-based – as a framework to understand team transitions and intervention effectiveness.

## Supplementary Information

Below is the link to the electronic supplementary material.

Supplementary Material 1

Supplementary Material 2

## Data Availability

Due to restrictions defined in the approved ethics application for this study, the datasets generated and analysed during this study cannot be shared with third parties. Data sharing is therefore not permitted.

## Abbreviations

SIT: Social Identity Theory
