# Supplementary material for: Reconstructing a typology of approaches to navigating diversity: a comparative qualitative study of German nursing teams
Source: BMC Nurs. 2026 Jun 9;25:521. doi: 10.1186/s12912-026-04854-y (PMC13248421; doi:10.1186/s12912-026-04854-y)
Supplement: Supplementary file 1 — Supplementary Material 1 [file 12912_2026_4854_MOESM1_ESM.pdf]

Interview guide for non-managerial nursing staff (English version)

0) Formalities

- Thank you for taking the time to participate in this interview!
- Brief introduction of the interviewer and brief presentation of the project.
- The contents of the interview will remain confidential.
- We will not share any personal information with colleagues or supervisors.
- Do you agree to us recording the interview? The recording can also be paused during the interview.
- The audio recording will be transcribed and then deleted. The transcript will be pseudonymised so that no conclusions can be drawn about individuals or institutions.
- Important: There are no right or wrong answers. What matters to us is your perspective. If you have no further questions and agree, I will now start the tape recording.

1) Open (narrative) introductory question

a) First, I would like to ask you to tell me a little about your background: How did you come to work here as a nurse today?

b) Can you tell me briefly about your working day yesterday?

  i) To what extent was it a normal or unusual working day?

2) Composition of the team

a) How would you describe the composition of the team here on the ward?

b) How would you describe your own role here in the team?

  i) What are your responsibilities in the team/on the ward?

3) Cooperation on the ward

a) Looking back over the last six months, can you remember an incident on the ward

  i) that particularly sticks in your mind?

  ii) where cooperation worked particularly well?

  iii) where cooperation did not work so well?

  iv) How did this affect patient care in each case?

b) While observing everyday life on the ward, I noticed the following: [insert aspect of participant observation]. How do you see this from your perspective?

c) Where, when and in what context do you normally discuss matters

  i) with your colleagues?

  ii) with your superiors?

d) What do you think: How would you describe the situation/collaboration here on the ward from the perspective of the senior nursing staff?

e) What do you think: How would you describe the collaboration with the nursing staff from the perspective of the medical staff?

4) Patients and relatives

a) What role do relatives play in your daily work?

i) Do you have a specific example?

ii) How does cooperation with relatives affect patient care?

5) External environment
   a) There is currently a lot of change and upheaval in nursing. How do you perceive the current developments – in nursing in general or specifically here on site?

6) Questions about the future
   a) If you could put together your dream team, what would it look like?
   b) In your opinion, what should definitely not be changed on the ward?
   c) What would be the first sign that working conditions have improved/deteriorated?

7) Conclusion:
   Is there anything else we haven't discussed yet that would be important to mention?

8) Follow-up questions (if not already discussed)
   a) How long have you been working in nursing?
   b) How long have you been working on this ward?
   c) What training do you have?
   d) In which country did you complete your training/studies?
   e) In your opinion, how does diversity in your nursing team influence cooperation and patient care?
