# Supplementary material for: Reconstructing a typology of approaches to navigating diversity: a comparative qualitative study of German nursing teams
Source: BMC Nurs. 2026 Jun 9;25:521. doi: 10.1186/s12912-026-04854-y (PMC13248421; doi:10.1186/s12912-026-04854-y)
Supplement: Supplementary file 2 — Supplementary Material 2 [file 12912_2026_4854_MOESM2_ESM.pdf]

Interview guide for nursing managers (English version)

0) Formalities

- Thank you for taking the time to participate in this interview!
- Brief introduction of the interviewer and brief presentation of the project.
- The contents of the interview will remain confidential.
- We will not share any personal information with colleagues or supervisors.
- Do you agree to us recording the interview? The recording can also be paused during the interview.
- The audio recording will be transcribed and then deleted. The transcript will be pseudonymised so that no conclusions can be drawn about individuals or institutions.
- Important: There are no right or wrong answers. What matters to us is your perspective. If you have no further questions and agree, I will now start the tape recording.

1) Open (narrative-generating) introductory question

a) To begin with, I would like to ask you to tell me a little about your career: you are working here today in this management position. How did that come about?

b) Can you tell me briefly about your working day yesterday?

  i) To what extent was it a normal or unusual working day?

2) Composition of the nursing teams

a) How would you describe the composition of the team on your ward?

b) What influence do you have on the composition of the team?

3) Cooperation on the ward

a) When you think back on the last six months,

  i) can you think of an event on your ward that particularly sticks in your mind? What role did nursing play in this?

  ii) can you think of an event at management level that particularly sticks in your mind? What role did you play in this?

b) When I was observing everyday life on the ward here, I noticed the following: [insert aspect of participant observation]. How do you see this from your perspective?

c) How are you specifically involved in everyday nursing care on the ward?

d) Where, when and in what context do you usually discuss matters

  i) with the nursing staff on the ward?

  ii) with the medical staff?

  iii) with the hospital management?

  iv) Can you outline the cooperation between the ward managers?

e) How would you describe the situation/cooperation on your ward?

  i) within nursing?

  ii) the cooperation between nursing staff and medical staff?

  iii) In your opinion, how does the diversity of the team influence cooperation?

4) Patients and relatives

a) What role do relatives play in your everyday work?

i) Do you have a specific example?

ii) How does cooperation with relatives affect patient care?

5) External environments:

a) A lot is happening in nursing at the moment, and many things are in a state of flux.

i) How do you perceive the current developments?

ii) How is the nursing situation changing here locally at the moment?

6) Questions about the future

a) If you could put together your dream team for your ward, what would it look like?

b) Imagine you had complete freedom to design your team. What would you change first?

c) What would be the first sign that working conditions in nursing had improved or deteriorated?

7) Conclusion:

Is there anything else we haven't discussed that would be important to mention?
